# Defining the Need for Causal Inference to Understand the Impact of Social Determinants of Health: A Primer on Behalf of the Consortium for the Holistic Assessment of Risk in Transplantation (CHART)

**DOI:** 10.1097/AS9.0000000000000337

**Published:** 2023-09-27

**Authors:** Nrupen A. Bhavsar, Rachel E. Patzer, David J. Taber, Katie Ross-Driscoll, Rhiannon Deierhoi Reed, Juan C. Caicedo-Ramirez, Elisa J. Gordon, Roland A. Matsouaka, Ursula Rogers, Wendy Webster, Andrew Adams, Allan D. Kirk, Lisa M. McElroy

**Affiliations:** From the * Department of Medicine, Duke University School of Medicine, Durham, NC; † Department of Biostatistics and Bioinformatics, Duke University School of Medicine, Durham, NC; ‡ Department of Surgery, Emory University School of Medicine, Atlanta, GA; § Department of Surgery, Medical University of South Carolina, Charleston, SC; ‖ Department of Surgery, The University of Alabama at Birmingham, Birmingham, AL; ¶ Department of Surgery, Northwestern University Feinberg School of Medicine, Chicago, IL; # Department of Surgery, Vanderbilt University Medical Center, Nashville, TN; ** Department of Surgery, Duke University School of Medicine, Durham, NC; †† Department of Surgery, University of Minnesota Medical School, Minneapolis, MN.

**Keywords:** causal inference, epidemiology, social determinants of health, transplantation

## Abstract

**Objective:**

This study aims to introduce key concepts and methods that inform the design of studies that seek to quantify the causal effect of social determinants of health (SDOH) on access to and outcomes following organ transplant.

**Background:**

The causal pathways between SDOH and transplant outcomes are poorly understood. This is partially due to the unstandardized and incomplete capture of the complex interactions between patients, their neighborhood environments, the tertiary care system, and structural factors that impact access and outcomes. Designing studies to quantify the causal impact of these factors on transplant access and outcomes requires an understanding of the fundamental concepts of causal inference.

**Methods:**

We present an overview of fundamental concepts in causal inference, including the potential outcomes framework and direct acyclic graphs. We discuss how to conceptualize SDOH in a causal framework and provide applied examples to illustrate how bias is introduced.

**Results:**

There is a need for direct measures of SDOH, increased measurement of latent and mediating variables, and multi-level frameworks for research that examine health inequities across multiple health systems to generalize results. We illustrate that biases can arise due to socioeconomic status, race/ethnicity, and incongruencies in language between the patient and clinician.

**Conclusions:**

Progress towards an equitable transplant system requires establishing causal pathways between psychosocial risk factors, access, and outcomes. This is predicated on accurate and precise quantification of social risk, best facilitated by improved organization of health system data and multicenter efforts to collect and learn from it in ways relevant to specialties and service lines.

## Abstract 2

Mini abstract

This article aims to introduce key concepts that inform the design of studies to quantify the causal effect of social determinants of health (SDOH) in access to and outcomes after organ transplant. To rapidly advance research at the intersection of SDOH and clinical outcomes in organ transplant, investigators must consider biases arising from socioeconomic status, race/ethnicity, and incongruencies in language between the patient and clinician at the time of study design. Transplant is an exemplar model, but the approaches reviewed can broadly be applied to other surgical and nonsurgical fields.

## INTRODUCTION

Social determinants of health (SDOH) weigh heavily in transplant candidacy as they are thought to reflect social risk, which in turn is thought to influence access to transplant and post-transplant outcomes. Research to date has characterized associations of SDOH with disparities along the continuum of end-stage organ disease and transplant care.^1,2^ Yet, the causal pathways between social risk and transplant outcomes are incompletely described and poorly understood. This is, in part, because the complex interactions between patients, their neighborhood environments, the transplant care system, and structural factors that impact access and outcomes (eg, discrimination and racism) are poorly captured in current data systems. It may also be due to a lack of adherence to principles of causal inference in research study design and subsequent failure to utilize appropriate methods to account for these complex interactions, thereby limiting the growth of cumulative scientific knowledge over time. The goal of this article is to introduce concepts and methods fundamental to the design of studies that aim to quantify the causal effect of SDOH on process and clinical outcomes in organ transplant. We present examples in clinical transplant research that necessitate causal inference methods and highlight areas vulnerable to bias.

## FUNDAMENTALS OF CAUSAL INFERENCE

The fundamental goal of biomedical research is to estimate the direct effect of an exposure or treatment on an outcome. The associations between social factors and health are complex and can be mediated by multiple factors, some of which are challenging to define (eg, adverse neighborhood environments), while others (eg, resilience and discrimination) are infrequently or poorly measured. Randomized controlled trials (RCT) are the gold standard study design to estimate the causal impact (ie, causal estimand) of a treatment or intervention on an outcome. However, due to practical or ethical constraints, RCTs are often infeasible, and observational data must be used to infer causality. As such, observational data has long been used to approximate the causal effects of SDOH on access to transplants, as well as post-transplant clinical outcomes.

### Potential Outcomes Framework

One approach to structure causal inference is through the potential outcomes framework, which formally defines the causal effect of interest before analysis is initiated.^3,4^ In the potential outcomes framework, causality is defined by what would have happened if, possibly counter to fact, the same patients were assigned to 2 different treatment options (or exposure conditions). Such a contrast in outcomes constitutes the causal effect of the 2 treatments (or exposures). In the real world, outcomes can only be observed in 1 treatment group; the outcome corresponding to the treatment that a person does not receive is called the counterfactual outcome. Since counterfactuals are unobservable, comparator individuals who were not assigned the said treatment are relied upon to estimate the causal effect of the treatment.

An example of an ideal (but impossible) causal framework would be one that measures the causal impact of poverty on transplant allograft rejection. To do this, we would need to identify a person who experienced poverty and for the same person imagine what would have happened had they not experienced poverty. Because one of these outcomes is unobservable, we instead identify other individuals who did not experience poverty but are similar in all other ways to the person in our study who did experience poverty. In addition to the impossibility of assigning the same person to poverty and the alternative exposure (ie, counterfactual), it would be unethical (or flat-out impossible) to randomize someone to experience poverty, or randomly remove certain patients from poverty. Nevertheless, using observational data, we can measure the causal impact of poverty on transplant allograft rejection. We first identify impoverished individuals and quantify their risk of experiencing allograft rejection. Then, we quantify the risk for allograft rejection in the same individuals had they not experienced poverty, by leveraging the information from similar individuals who did not experience poverty (counterfactual analysis). However, comparator groups will not always have identical demographic, social, and clinical characteristics as the poverty-exposed group. When these differences in characteristics between the exposed and comparator groups are also associated with the outcomes, they may confound, that is, muddied the association of interest. Moreover, they may be in the causal pathway between the exposure and outcome, and thus function as mediators (as opposed to direct factors) of the exposure (or treatment)—outcome relationship.

There are key assumptions that must be met to use the potential outcomes framework.^5^ These include:

1. *Stable unit value assumption (SUTVA*): for each treatment considered, there is only 1 version of said treatment, which corresponds to a specific potential outcome. Moreover, the potential outcomes of an individual are not affected by the treatment received by other individuals. The SUTVA assumption is violated, for instance, when there is a possibility for interference, contagion, or spillover effect (eg, through an influenza vaccination campaign where the vaccination status of 1 individual may affect the risk of another individual being infected).
2. *Consistency*: an individual’s observed outcomes coincide with the potential outcome for the treatment the individual actually received. (At times consistency is considered part of SUTVA.).
3. *The positivity assumption*: given an individual’s measured characteristics, the probability of receiving a treatment is nonzero.
4. *Exchangeability* (ie, no unmeasured confounders): condition on the characteristics that may influence the potential outcomes, individuals who received one treatment are exchangeable (similar on average) with those who received an alternative treatment.

In transplantation, however, these assumptions can be violated. If the outcome of interest is being added to the waitlist, SUTVA is not violated because one individual being waitlisted does not impact the probability that another individual is waitlisted. However, one individual receiving an organ can ipso facto prevent another individual from getting an organ, which violates the positivity assumption or the SUTVA. There are also multiple types of treatments a patient can receive (eg, living vs deceased organ donor, Blood group compatible vs incompatible) which would violate the consistency assumption if not accounted properly. Finally, there are unmeasured factors (eg, physician bias and patient engagement) that are intrinsically related to and can profoundly affect the treatment-outcome relationship (and thus violating the exchangeability assumption).

### Directed Acyclic Graphs

**FIGURE 1.**
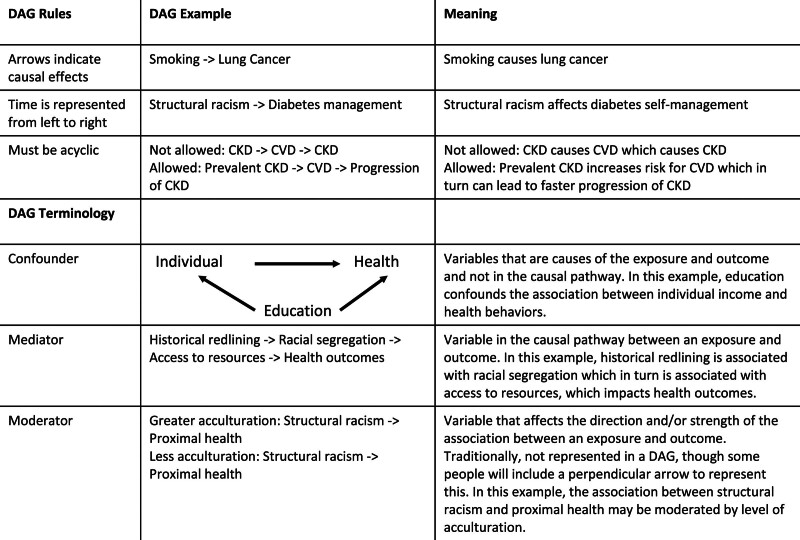
Directed Acyclic Graphs: Rules and Terminology

**FIGURE 2.**
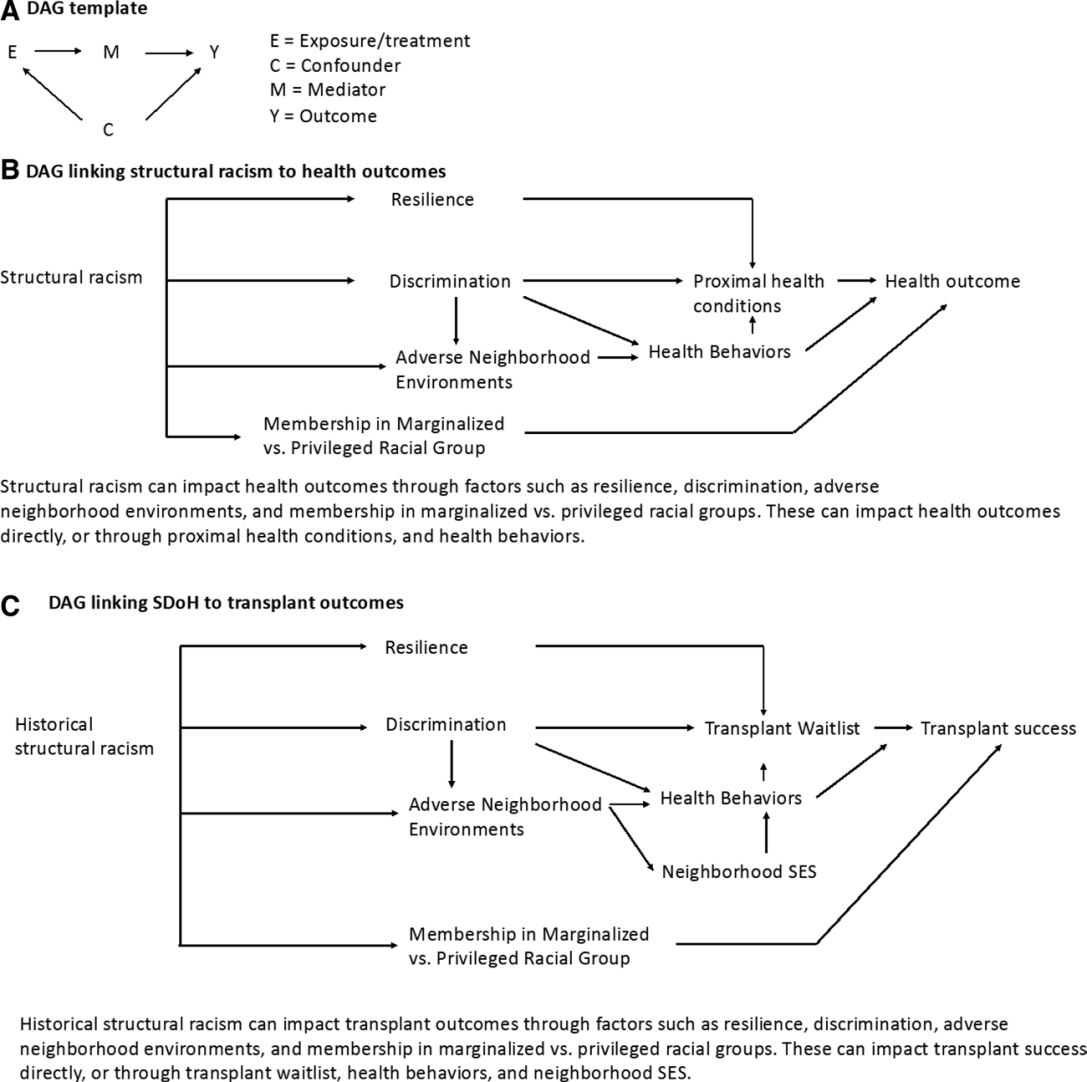
Examples of Directed Acyclic Graphs (DAGS). A, DAG template. B, DAG linking structural racism to health outcomes. C, DAG linking SDOH to transplant outcomes. SDOH, social determinants of health.

Conceptualizing these relationships in a causal framework when designing a study and conducting the analysis with methodologic rigor is thus paramount. One approach is through direct acyclic graphs (DAGs).^6,7^ DAGs allow conceptualization and provide a visual representation of the relationship between exposures or treatments, outcomes, confounders, and mediators. They also help understand the potential interplay among variables, identify sources of bias and ways to adjust for them, or simply uncover a number of phenomena that may cloud the relationship between exposure and outcomes (beyond confounders and mediators), including selection bias, collider bias, censoring, and so on.^8–10^ Finally, based on the scientific questions of interest, the use of DAGs helps bring clarity on what type of analyses can adequately assess the effect of treatment or exposure, what variables should (or must not) be adjusted for, and possible ways to adjust for them. Others have provided a robust foundation and overview to support the use of DAGs in a variety of clinical disciplines. Through the process of creating DAGs, variables are also identified that are not measured or incompletely measured, termed latent variables. In the absence of complete and accurate measures, different approaches can be applied to compensate for measurement flaws. These can include using proxy variables, leveraging specific analytical approaches (eg, instrumental variables), or running sensitivity analyses. While a comprehensive discussion of DAGs is important, it is beyond the scope of this article. There are, however, fundamental rules worthy of mention when creating a DAG (Fig. 2). Figure 1 provides a standard template for creating DAGs (Fig. 2A) and DAGs more closely related to social epidemiology (Fig. 2B) and transplant equity research (Fig. 2C).^7^

## CONCEPTUALIZING SDOH FOR CAUSAL INFERENCE

### Theory of Fundamental Causes

Important to the development of strategies to address inequities in access to transplant, organ allocation, and outcomes is understanding the fundamental causes of health inequities. The theory of fundamental causes states that there are 4 essential elements of health inequalities.^11^ These include (1) the social cause that influences multiple disease outcomes; (2) the social cause that affects outcomes through multiple risk factors; (3) the social cause that involves access to resources used to avoid risks or minimize consequences of disease once it occurs; and (4) the reduction of the association between fundamental cause and health over time via replacement of intervening mechanisms. As an example, income is a fundamental cause of health inequities because the association between income and health persists despite changes to mediating variables that link income with health.

Individuals and groups with knowledge/education, money, power, prestige, and beneficial social connections leverage these factors to derive good health outcomes, which further causes health inequities to persist over time. At the individual level, flexible resources are conceptualized as a ‘cause of causes or risk of risks’ that shape individual behavior.^11^ As an example, resources such as knowledge/education and money allow an individual to get support for health-enhancing activities (eg, patients with the ability to travel to and be listed at more than 1 transplant center). Many of the individual determinants of health are influenced by contextual-level factors. These can be at the level of families, congressional blocks, and formal (employer, trade union) or informal (social network) groups. A person can afford to live in higher socioeconomic status neighborhoods where collectively the neighborhood can address factors that adversely affect health, such as noise, violence, and pollution. It is important to generate hypotheses, design research studies and develop interventions that address the fundamental causes at the appropriate level. Strategies can include reducing resource inequalities (eg, minimum wage, housing for homeless/low income, cap gain/estate taxes, parenting leave, and social security), contextualizing risk factors (eg, improving access to neighborhood environments that make healthful foods unavailable), empowering communities, making interventions passive (eg, free parking at hospitals and clinics instead of advocating for public transportation use, health screenings at schools/churches instead of at private practices.).

### Measuring Individual-Level Social Determinants of Health

A central consideration in designing epidemiologic studies is defining the variables of interest. SDOH are measured by variables that reflect social constructs such as race, income, and insurance status. Often, there is a misunderstanding of what a social construct represents, and/or there is heterogeneity in the definition and measurement of social constructs.^12^ For example, while poverty is a quantifiable SDOH through income, which is routinely collected by the census, other constructs such as trust, resilience, discrimination, or clinician communication behaviors are not as easily or reliably quantified.^13^ Race has been, and continues to be, used as a covariate in inferential studies along with demographic, biologic/clinical, and psychosocial variables. At times, as others have noted, these variables are in the pathway between race and a health outcomes (for example skin cancer and melanin).^14^ However, in a causal inference/potential outcomes framework, including race as an exposure is problematic. Rarely is there a biologic cause linking race with an outcome; often the underlying construct of interest is not race but other latent constructs, and race is thus used as a proxy for a complex array of social constructs.^15^ Research that aims to study the association between racism and health has historically used race as a proxy for racism, but this approach has several methodological flaws and can lead to biased inferences. Important and undermeasured constructs include systematic and structural racism which are “forms of racism that are pervasively and deeply embedded in systems, laws, written or unwritten policies, and entrenched practices and beliefs that produce, condone, and perpetuate widespread unfair treatment and oppression of people of color, with adverse health consequences.”^11^ One recent example of this is race-based estimates of kidney function, which systematically impacts individuals racialized as “Black” by delaying the time to transplant referral and waitlisting by an estimated period of 1.9 median years. The recent exposure of this upstream barrier and the decision by the US Organ Procurement and Transplantation Network to abandon race-based kidney function estimating equations provides an exemplar of how a focus on justice, reform, and repair can advance our efforts to pursue equity. Other examples of latent constructs include concepts such as trust, discrimination, self-actualization, and resilience (among others) that are rarely collected during clinical care or in the electronic health record. SDOH data vary in their completeness and accuracy across data sources and vary in their ability to accurately reflect social constructs. As an example, individual-level SDOH data has historically had extensive missingness in the EHR and instead, neighborhood-level SDOH has been used as a proxy. In the context of transplant equity research, collecting and defining these latent constructs is paramount to developing strategies to mitigate the inequities they perpetuate.

### Measuring Neighborhood-Level Social Determinants of Health

Due to the challenges of collecting individual-level SDOH data and defining latent constructs, summary (neighborhood-level) indices are often used to quantify exposure to social and environmental contextual factors. Multiple indices have been proposed, including the Area Deprivation Index, the Social Vulnerability Index, and the Racial Equity Index, to quantify socioeconomic status at the neighborhood level and identify geographic patterns of social risk. These indices use publicly available population-level data to identify place-based patterns of social risk that impact health. The information from these summary indices can help identify at-risk communities and generate hypotheses related to environmental exposures and care delivery systems. However, there are notable methodological challenges with using these indices, including the fact that (1) they are updated infrequently; (2) the underlying data used to create them can be proprietary; (3) important underlying constructs such as structural racism may not be measured; (4) they were not created to study health disparities; (5) they can promote the ecological fallacy whereby inferences at the neighborhood level are applied to individuals, leading to inappropriate or even harmful research or treatment decisions.

## APPLIED EXAMPLES LINKING SDOH TO INEQUITIES IN ORGAN TRANSPLANT

The fundamental questions before transplant selection committees are related to waitlist mortality and post-transplant patient and graft survival. The following cases illustrate the bias that can be introduced from spurious associations.

### Case 1

AB is a 72-year-old non-Hispanic white male with end-stage liver disease due to nonalcoholic steatohepatitis. His current model for end stage liver disease is 26. He has insulin-dependent diabetes as well as coronary artery disease. He has 2 coronary stents in place, an ejection fraction of 40%, and a recent cardiac stress test showed no signs of inducible ischemia. His body mass index (BMI) is 38; he is a farmer and still works part-time. He has a son who lives with him, multiple family members for support, significant savings through a family trust, and private insurance. He lives 3 hours from his transplant center in a rural community.

In the case of AB, his occupation as a farmer is falsely thought to predict resilience, which is a latent construct. This contrasts with frailty which can be quantified via validated measures. AB may be falsely thought to be less frail than would be quantified by Karnofsky score or 6-minute walk test due to his occupation, despite more advanced age and more extensive medical comorbidities.

### Case 2

CD is a 38-year-old non-Hispanic white female with hypertension-related kidney disease. She has been on dialysis for 3 years. She has no additional medical comorbidities, and her surgical history is significant for an open cholecystectomy. Her BMI is 24 and she works part-time as a customer service provider from home. She has multiple family members for support, but all have minimum-wage jobs without allowed time off. She lives in a 2-bedroom home with 4 adults, has low income, no savings, and Medicaid insurance. She lives 30 minutes from her transplant center in a rent-stabilized building within a high socioeconomic status community.

In the case of CD, her multigenerational poverty, as indicated by multiple family members with minimum-wage jobs, is falsely thought to predict nonadherence which is a latent construct. CD may falsely be thought to be less adherent to follow-up care than would be quantified by communication with her primary nephrologist and dialysis center about her engagement in care, review of visit cancellations and no-shows, and medication adherence measures. Despite her poverty, she may have a clear, objective record of consistent adherence to medical care.

### Case 3

EF is a 27-year-old White Hispanic male with diabetes and hypertension-induced kidney disease. His current Hb A1c is 7.8. He is on dialysis via a vascular catheter after failing home peritoneal dialysis due to multiple episodes of peritonitis. His calculated panel reactive antibody is 20. His BMI is 32 and works from home as a phone customer service representative. He lives with his parents due to a neurodevelopmental delay and is non-English speaking. He has Medicaid insurance. His parents are in good health and consistent caregivers with significant income and savings. EF was not considered as a good candidate for a transplant because the clinicians had a difficult time communicating with him and his parents. Additionally, there are concerns about noncompliance based on dialysis run sheets.

In the case of EF, incongruent language and cultures manifested as limiting factors and ruled out the patient for a transplant. While clinicians may view these as limiting factors, the parents’ good health, history of caregiving, and financial resources, in addition to potential protective cultural factors, may lead to better outcomes for the patient.

## USE OF CAUSAL INFERENCE TO UNDERSTAND THE IMPACT OF SOCIAL RISKS IN TRANSPLANT POPULATIONS

It is widely accepted that SDOH drives overall health status. However, significant work remains to elucidate the causal mechanisms by which this occurs in transplantation. To date, the work in transplant disparities, largely characterizes associations through retrospective examination, relying on proxy data, and oversimplifying the complex interactions between patients, environments, clinicians, and the healthcare system. Studies focusing on the identification of patient-level factors can inadvertently shift the blame for poor outcomes onto patients themselves without accounting for the larger context of structural barriers and complexities of clinician-patient interactions. As a transplant community (ie, clinicians and researchers), we have not yet quantified social risk in a way that allows consistent assessment of its impact on patient and graft survival. In the same regard, we also fail to understand what effects are suffered by patients with social complications who may have good long-term clinical outcomes. Many of the proxy variables that reflect SDOH cannot be established as a direct cause of any clinical outcome, and latent and mediating variables that tie SDOH with health inequities remain unmeasured.

To establish causal relationships between social risk and outcomes in transplant, research methods must expand beyond merely broad associations and study fundamental causes of inequities that have been measured for more than 3 decades. In addition to improved data architecture, modern analytic approaches such as hierarchical models can characterize health inequities by quantifying the impact of factors at multiple levels (eg, patient, health system, community). Propensity score methods can help make more accurate causal inferences by addressing imbalances between the exposure/treatment group and the counterfactual group. Robins’ generalized methods (ie, g methods) can provide more flexibility in the context of time-varying treatments and confounders than traditional regression methods (eg, linear, logistic, and Cox regression) when quantifying differences or ratios in average potential outcomes.^16,17^ To do so, concerted efforts are required by interdisciplinary teams with methodological expertise in biostatistics, clinical trials, ethics, epidemiology, informatics, and implementation science to develop novel approaches to curating clinical, health system, and SDOH data.

Identifying causal relationships between social risk and adverse clinical outcomes can demonstrate the direct and critical impact of SDOH in organ transplantation. Multi-level frameworks for research and examination of health system data through multicenter research networks can help us understand (and mitigate) both how social risks impact patient and graft survival and how social complications occur in patients with optimal graft survival. Measuring and characterizing center- and system-level drivers of inequities in access to transplant will allow the development and implementation of targeted interventions that improve equity in access to transplant through inclusive and objective systems and processes of care. Collaborations across different institutions serving distinct populations and geographic regions in this vein have the potential to impart foundational change to the transplant selection process, leading to improved equity in access to care, increased transparency in the transplant selection process, and improved value for patients and clinicians. This work is critical for progress towards health equity not just in transplant, but in all complex multispecialty disciplines that serve patients with multiple chronic conditions. Several registry networks are already well-established across surgical specialties and could serve as the foundation for enhanced study of SDOH using health system data The framework and approaches described here can also be extended to nonsurgical fields that may have similar health inequities, data curation challenges, and complex care pathways.
